# Glycerophosphoinositol modulates FGA and NOTCH3 in exercise-induced muscle adaptation and colon cancer progression

**DOI:** 10.3389/fphar.2024.1430400

**Published:** 2024-07-26

**Authors:** Hongbiao Luo, Wei Luo, Ning Ding, Huimin Zhu, Jiahui Lai, Qingzhu Tang, Yongheng He

**Affiliations:** ^1^ Department of Anorectal Surgery, Chenzhou NO. 1 People’s Hospital, Chenzhou, Hunan, China; ^2^ Hunan University of Chinese Medicine, Changsha, Hunan, China; ^3^ The Second Clinical Medical College of Nanchang University, The Second Affiliated Hospital of Nanchang University, Nanchang, Jiangxi, China; ^4^ Department of Critical Care Medicine, Chenzhou NO. 1 People’s Hospital, Chenzhou, Hunan, China; ^5^ The Third Hospital, Hebei Medical University, Shijiazhuang, China; ^6^ Affiliated Hospital of Hunan Academy of Traditional Chinese Medicine, Changsha, Hunan, China

**Keywords:** FGA, Notch3, PTMs, colon cancer, prognosis, gene expression

## Abstract

**Objectives:**

Fibroleukin (FGA) and NOTCH3 are vital in both exercise-induced muscle adaptation and colon adenocarcinoma (COAD) progression. This study aims to elucidate the roles of FGA and NOTCH3 in phenotypic variations of striated muscle induced by exercise and in COAD development. Additionally, it seeks to evaluate the prognostic significance of these proteins.

**Methods:**

Gene Set Variation Analysis (GSVA) and protein-protein interaction (PPI) network analysis were employed to identify differentially expressed genes (DEGs). Molecular docking studies were conducted to assess the binding affinities of 39 compounds to the NOTCH3 protein. *In vitro* assays, including mobileular viability, gene expression, and apoptosis assays, were performed to evaluate the effects of glycerophosphoinositol on FGA and NOTCH3 expression. Additionally, copy number variation (CNV), methylation status, and survival analyses were conducted across multiple cancers types.

**Results:**

The NOTCH signaling pathway was consistently upregulated in exercise-induced muscle samples. High NOTCH3 expression was associated with poor prognosis in COAD, extracellular matrix organization, immune infiltration, and activation of the PI3K-Akt pathway. Molecular docking identified gamma-Glu-Trp, gamma-Glutamyltyrosine, and 17-Deoxycortisol as strong binders to NOTCH3. Glycerophosphoinositol treatment modulated FGA and NOTCH3 expression, influencing cell proliferation and apoptosis. CNV and methylation analyses revealed specific changes in FGA and NOTCH3 across 20 cancers types. Survival analyses showed strong associations between FGA/NOTCH3 expression and survival metrics, with negative correlations for FGA and positive correlations for NOTCH3.

**Conclusion:**

FGA and NOTCH3 play significant roles in exercise-induced muscle adaptation and colon cancer progression. The expression profiles and interactions of these proteins provide promising prognostic markers and therapeutic targets. These findings offer valuable insights into the post-translational modifications (PTMs) in human cancer, highlighting novel pharmacological and therapeutic opportunities.

## Introduction

Colorectal cancer (CRC), a leading cause of cancer-related mortality worldwide, is characterized by a complex interaction of genetic and environmental factors (Miles et al., 2015; Baidoun et al., 2021). Among these factors, physical exercise has been increasingly recognized for its potential to mitigate CRC risk and progression, as well as to enhance muscle health (Arena and McNeil, 2017). However, the molecular mechanisms underlying these effects remain to be fully elucidated.

Exercise-associated research provides insights for multi-omics evaluations (Chen et al., 2022a; Luo et al., 2022; Khan et al., 2023; Du and Liu, 2024; Wan et al., 2024), new therapeutic approaches and classification systems (Hussein and Rubenb, 2022; Lu et al., 2023; Torres-Rosas et al., 2023; Chen et al., 2024). Exercise-induced adaptations in striated muscle often involve complex molecular pathways, with post-translational modifications (PTMs) playing a crucial role in regulating key proteins (Ohlendieck, 2013; Solís and Russell, 2021). Similarly, in the progression of colon adenocarcinoma (COAD), PTMs can alter protein function and interaction networks, affecting tumor growth, immune response, and patient prognosis (Wang Y-W. et al., 2023).

Natural products hold potential for cancer treatment (Barreto and Jandus, 2022), translating into preventive and therapeutic options for CRC due to similarities in drug mechanisms and bioinformatics approaches (Feng et al., 2022; Simani et al., 2023; Xia et al., 2023; Xu et al., 2024). For instance, phenolic compounds in lentils exhibit significant antioxidant capacity, highlighting the importance of bioactive compounds related to exercise-induced muscle adaptation (Xia et al., 2023). Guishao tea extract inhibits gastric cancer growth (Liu et al., 2022), demonstrating the potential value of natural products in cancer therapy. Machine learning applied to cancer biomarker discovery can notably enhance early detection of CRC, highlighting the significance of computational tools (William et al., 2023; Zhong et al., 2023). This approach is vital for the detection and treatment of colorectal cancer (Mancheng and Ssas, 2023).

The findings of this study aim to improve our understanding of the molecular underpinnings of exercise-induced muscle adaptation and CRC development (Bonilla and Moreno, 2016). By identifying FGA and NOTCH3 as potential prognostic markers and therapeutic targets, our study could pave the way for developing personalized exercise regimens and targeted therapies for CRC patients. Furthermore, integrating systems biology approaches, including Gene Set Variation Analysis (GSVA) and protein-protein interaction (PPI) network analysis, will provide a more comprehensive view of the molecular landscape. This research could inform policy decisions promoting physical activity for cancer prevention and rehabilitation, ultimately improving patient care and quality of life.

## Materials and methods

### Data collection and identification of key genes and pathways

The study utilized several publicly available datasets: GSE213649 and GSE39582. We sourced skeletal muscle and colon data from GEO (Barrett et al., 2012), specifically using dataset GSE213649. Additionally, a cohort of locally advanced rectal cancer (LARC) patients was assembled, comprising resected tissues from 27 individuals. This included nine patients who achieved a complete pathological response (pCR), nine patients with no pathological response (npCR), and biopsy tissues from nine patients prior to undergoing neoadjuvant chemoradiotherapy (nCRT). The samples were categorised into skeletal muscle and colon groups, and differential analysis between exercising and non-exercising samples was conducted using the limma package. Significant differences were defined as |logFC| > 1 and *p*-value <0.05 (Ritchie et al., 2015). The results were visualized in volcano plots using the ggplot2 package (Wu et al., 2021). To identify exercise-related pathways, GSVA enrichment analysis was conducted separately on the skeletal muscle and colon groups using the GSVA package. Finally, key genes and pathways associated with exercise in both skeletal muscle and colon were identified through intersection.

### Molecular docking analysis

We downloaded 39 chemical compounds from the PubChem database using their CID numbers. These compounds were selected based on their known or potential roles in metabolic processes influenced by exercise. The three-dimensional structure of the NOTCH3 protein was obtained from the Protein Data Bank (PDB ID: specific PDB ID). The protein structure was prepared for docking by removing water molecules, adding hydrogen atoms, and optimizing the geometry using AutoDockTools. The 3D structures of the 39 chemical compounds were downloaded from PubChem. The preparation steps for each ligand were as follows:1. Energy minimization was performed using the MMFF94 force field in Avogadro software; 2. The ligands were converted to the PDBQT format required for docking using AutoDockTools. Molecular docking was carried out using AutoDock Vina. The docking grid was centered on the active site of the NOTCH3 protein, with dimensions set to ensure the entire binding pocket was covered. The exhaustiveness parameter was set to eight to ensure sufficient sampling of ligand conformations. Each ligand was docked to the NOTCH3 protein, and the binding affinity was calculated in terms of binding energy (kcal/mol). The lower the binding energy, the stronger the predicted binding affinity between the ligand and the protein. The top five chemical compounds displaying the strongest binding affinities (lowest binding energies) were identified. Detailed docking results were analyzed, and the binding poses were visualized using PyMOL software. Protein docking was performed using the GRAMM (Global RAnge Molecular Matching) web server (Katchalski-Katzir et al., 1992; Vakser, 1996; Tovchigrechko and Vakser, 2006; Singh et al., 2020). GRAMM systematically maps the intermolecular energy landscape by predicting a spectrum of docking poses corresponding to stable (deep energy minima) and transient (shallow minima) protein interactions. The docking grid was centered on the active site of the NOTCH3 protein. The dimensions were set to ensure the entire binding pocket was covered. Protein docking visualization was completed using PDBePISA (Proteins, Interfaces, Structures and Assemblies) (Krissinel, 2010).

### PPI and correlation analysis

Key genes were subjected to protein-protein interaction (PPI) analysis using the STRING platform. Subsequently, the relationships between significant pathways and genes were examined via Spearman correlation analysis.

### NOTCH3 gene landscape

The TIMER database [https://www.proteinatlas.org/] was employed to evaluate the differential expression of the NOTCH3 gene across various cancers and its correlation with immune cells. Tumor samples were categorized into high-expression and low-expression groups based on NOTCH3 expression levels, and prognostic differences between the two groups were analyzed. Additionally, the Human Protein Atlas (HPA) database was used to determine the tissue expression of NOTCH3 in bladder cancer.

### Single-gene enrichment analysis

Colon cancer data was extracted from the TCGA database (Tomczak et al., 2015). The limma package was employed to conduct differential analysis between the high- and low-expression groups of NOTCH3 within tumor tissues. Genes showing |logFC| > 0.5 and an adjusted *p*-value <0.05 were considered significantly differentially expressed (Ritchie et al., 2015).

### Immune infiltration

The estimate package was utilized to assess immune, stromal, and ESTIMATE scores for tumor tissues. Infiltration levels of 22 types of immune cells were determined using the Cibersort website (https://www.genecards.org/). Finally, the GEPIA website was leveraged to evaluate the correlation between NOTCH3 expression and immune checkpoint markers.

### Pan-cancer copy number variation and promoter methylation analysis

Copy number variation and DNA methylation data for various cancers were obtained from the TCGA database. The FGA and NOTCH3 copy numbers were extracted from different tumor tissues and categorized into amplification and deletion groups. Amplification and deletion rates were then calculated to assess the rates of FGA and NOTCH3 in various cancers. UALCAN (http://ualcan.path.uab.edu/analysis.html) was employed to explore promoter DNA methylation levels of FGA and NOTCH3 in both normal and pan-cancer tissues. DNA methylation maps for FGA and NOTCH3 across multiple cancer types were retrieved from the MethSurv database.

### Tumor prognosis analysis

Survival data were gathered from TCGA samples. Four indicators were used to assess the relationship between FGA and NOTCH3 expression and patient prognosis: overall survival (OS), disease-specific survival (DSS), progression-free interval (PFI), and disease-free interval (DFI). Survival analysis for each cancer type was conducted using the Kaplan-Meier method and log-rank test. Survival curves were plotted with the “survival” and “survminer” R packages, while the “forestplot” R package was used to elucidate the relationship between FGA and NOTCH3 expression and survival across cancers.

### Cell culture and transfection

The murine macrophage cell line RAW 264.7 (ATCC, Manassas, VA, USA) was maintained in DMEM supplemented with 10% fetal bovine serum (FBS), 100 μg/L streptomycin, and 100 IU/mL penicillin, at 37 °C in a 5% CO_2_ atmosphere. The SW620 cell line, procured from ATCC, was grown in DMEM (#06-1170-87-1A, Biological Industries, Israel) containing 10% FBS (#04-011-1A, Biological Industries, Israel), 100 U/mL penicillin, and 100 mg/mL streptomycin (#03-034-1B, Biological Industries, Israel). Incubation was performed in a Thermo Scientific incubator (USA) at 37 °C with 5% CO_2_. Wnt 5 (#sc-41112) and β-catenin (#sc-29209) siRNA, along with control siRNA (#sc-37007), were acquired from Santa Cruz Biotechnology (CA, USA). Plasmids for pcDNA-Wnt 5, pcDNA-β-catenin, and the control vector were sourced from Addgene (Cambridge, UK). SW620 cells were seeded at a density of 10^5^ cells per well in six-well plates and transfected with Lipofectamine 3,000 reagent (#L3000015, Invitrogen, CA, USA) following the manufacturer’s instructions. Transfections were performed using 50 nM siRNA or control siRNA, and complete culture medium was added after 6 hours, allowing cells to continue growing for another 12 h before being harvested for subsequent experiments (Sun et al., 2016).

### CCK8

The CCK-8 assay was performed as described previously. Cells were plated in 96-well plates with 1000 cells per well and incubated for 48 h under the indicated treatment. Cells were then incubated with CCK-8 for 4 h, and the OD value was measured at a wavelength of 490 nm.

### Flow cytometric apoptosis analysis

Cells (2 × 10^5^/sample) were incubated in complete medium at 37°C for 20 min, washed with PBS, and fixed in 100 µL of fixation buffer (eBiosciences, #420801) for 15 min at room temperature. They were then rinsed with PBS containing 1% BSA (AppliChem PanReac, #A6588) and treated with 10 µL of permeabilization buffer (eBiosciences, #421008) containing either mouse anti-Bax (B-9) (Santa Cruz Biotechnology Inc., #sc-7480) or rabbit anti-phospho-SHP-1 Tyr564 (Cell Signaling, #D11G5) antibodies for 1 h at room temperature. After washing twice with PBS containing 1% BSA, cells were incubated with 10 µL permeabilization buffer containing either Alexa Fluor anti-mouse-488 (Thermo Fisher Scientific, #A11001) or anti-rabbit-488 (Thermo Fisher Scientific, #A11008) secondary antibodies for 45 min. Cells were then washed with PBS containing 1% BSA, resuspended in 200 µL of PBS with 1% BSA, and analyzed via flow cytometry. To identify early apoptotic cells, cells were stained with FITC-labeled Annexin V (e-Bioscience, #88-8005–74) and propidium iodide (PI, 20 μg/mL, Biotium, #40017), followed by flow cytometry analysis. Mitochondrial membrane potential was measured using the fluorescent probe tetramethylrhodamine methyl ester (TMRM, Molecular Probes Europe BV). For this measurement, 1 × 10^6^ cells were suspended in 200 µL of RPMI-1640 without phenol red (Invitrogen srl), supplemented with 25 mM Hepes (pH 7.4) and 200 nM TMRM, and incubated at 37 °C for 20 min. Calcium ionophore A23187 (500 ng/mL, Sigma-Aldrich #C7522) was then added, followed by a 10-min incubation at 37 °C before flow cytometric analysis using a Guava Millipore cytometer. Flow cytometric data were analyzed using FlowJo (Tree Star, Inc.).

### qRT-PCR

Total RNA was extracted from gastric cancer cells using the Trizol reagent (15,596-018, Invitrogen), followed by reverse transcription using the M-MLV Reverse Transcriptase Kit (M1701, Promega). Quantitative PCR was then conducted using the SYBR reagent (RR420A, Takara). The mRNA levels of FGA and NOTCH3 were normalized to GAPDH expression.

### Statistical analysis

All statistical analyses were performed using R software e (version 4.0.2). Differences between two groups were assessed using the Student’s t-test. A *p*-value of less than 0.05 was considered statistically significant. For comparisons involving more than two groups, a one-way analysis of variance (ANOVA) followed by Tukey’s multiple comparisons test was applied.

## Results

### Identification of key genes and pathways

**FIGURE 1:**
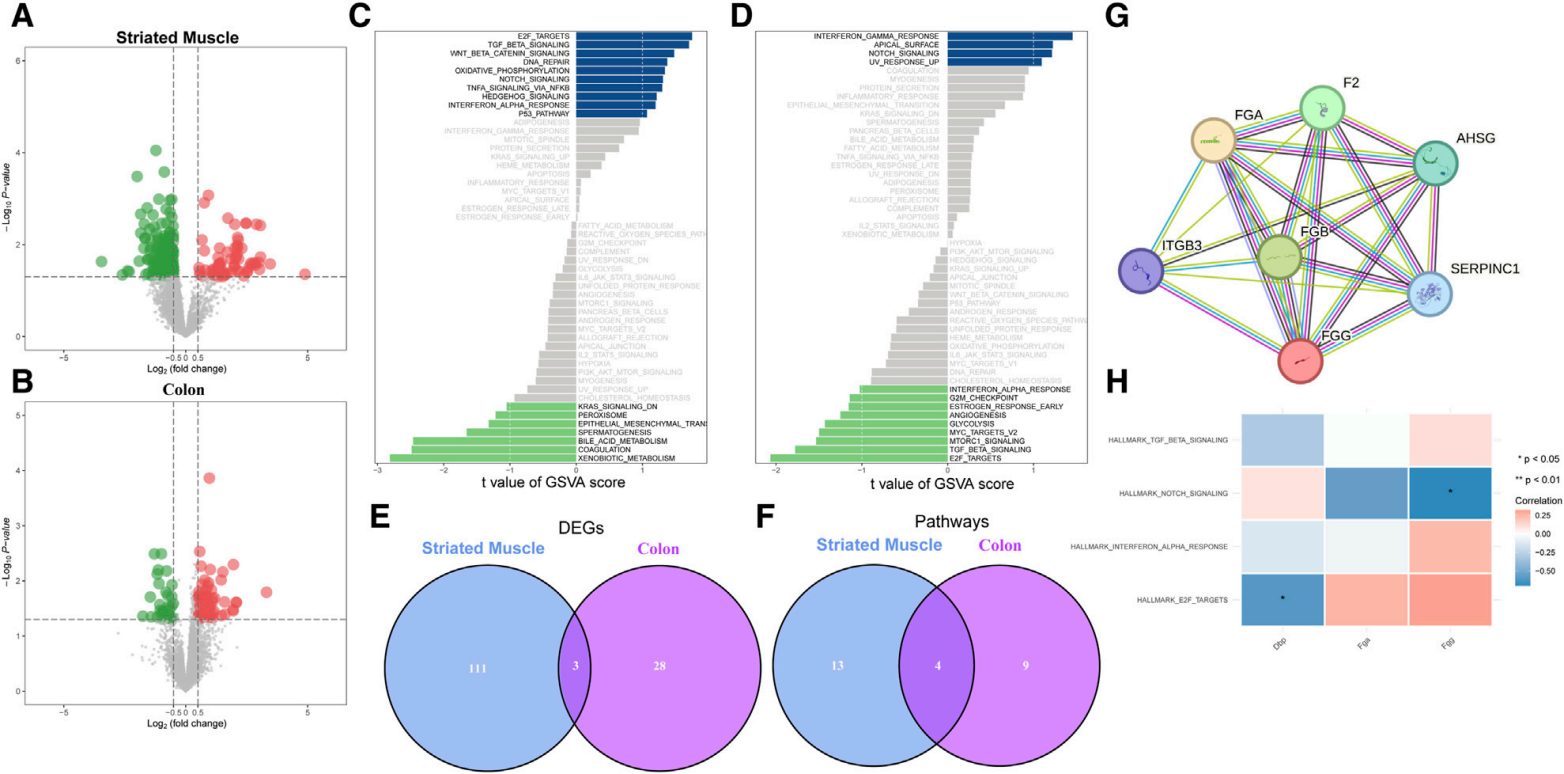
Identification of key genes and pathways. **(A)** Volcano plot showing differentially expressed genes (DEGs) in striated muscle tissue. Red and green dots indicate upregulated and downregulated genes, respectively. **(B)** Volcano plot of DEGs in colon tissue, with color coding similar to **(A)**. **(C)** Gene Set Variation Analysis (GSVA) reveals enriched pathways associated with muscle contraction in striated muscle. Positive and negative values on the *x*-axis represent upregulation and downregulation, respectively. **(D)** GSVA highlighting enriched pathways related to muscle contraction in colon tissue. **(E)** Venn diagram showing overlap of DEGs between striated muscle and colon tissues. **(F)** Venn diagram indicating overlap of muscle contraction-related pathways between striated muscle and colon tissues. **(G)** Protein-protein interaction (PPI) network analysis of key genes. Connections between nodes represent interactions. **(H)** Correlation analysis between key genes and muscle contraction-related pathways. Rows represent pathways, and columns represent genes. Blue and red indicate negative and positive correlations, respectively.

In the striated muscle group, a total of 114 differentially expressed genes (DEGs) were identified, with 56 upregulated and 58 downregulated in exercise samples (Figure 1A). In the colon group, 31 DEGs were found, including 21 upregulated and 10 downregulated genes in exercise samples (Figure 1B). Gene Set Variation Analysis (GSVA) revealed significant phenotypic differences between the exercise and non-exercise groups, with distinct pathways associated with muscle contraction (Figures 1C, D). Notably, three genes were found to be differentially expressed in both groups: FGG and FGA were upregulated in exercise samples, whereas DBP was downregulated (Figure 1E). Additionally, four pathways were consistently enriched across the two groups, with the NOTCH signaling pathway being the only one upregulated in exercise samples from both groups (Figure 1F). To investigate potential interactions among the identified key genes, a protein-protein interaction (PPI) network was constructed, showing significant relationships among FGG, FGA, and FGB (Figure 1G). Furthermore, correlation analysis revealed a negative relationship between the NOTCH signaling pathway and both FGG and FGA expression (Figure 1H). These findings suggest that the regulation of FGG and FGA expression by the NOTCH signaling pathway may represent a critical activation mechanism in exercise samples, contributing to the observed phenotypic differences between the exercise and non-exercise groups.

### Function of NOTCH3 in colon cancer

**FIGURE 2:**
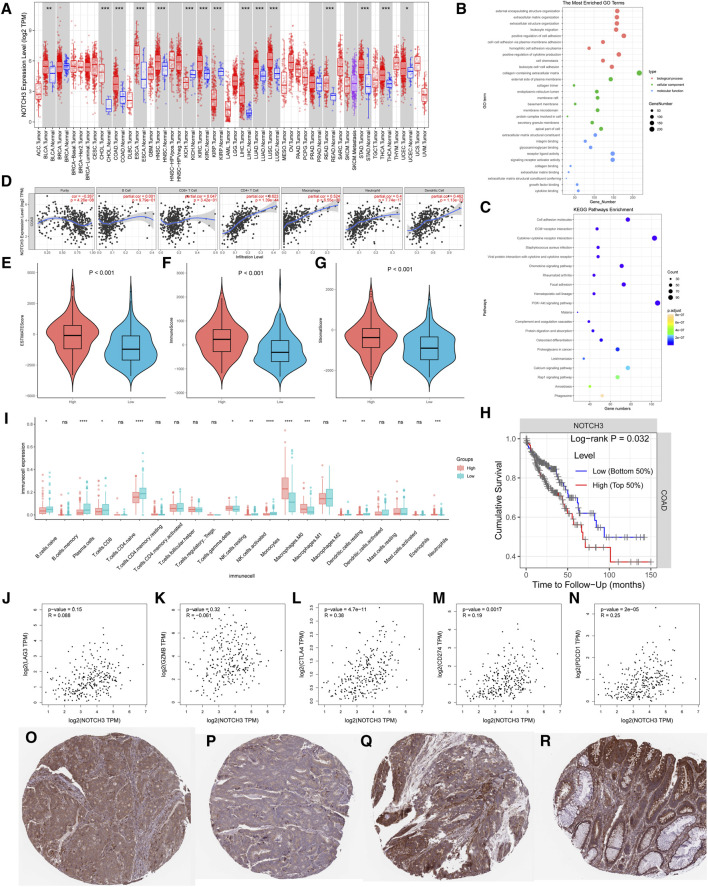
Analysis of key genes and their impact on pathways and survival. **(A)** Expression profile of the NOTCH3 gene across multiple cancer types. The red and blue boxes represent differential expression levels, with statistical significance indicated for each comparison. **(B)** Gene Set Enrichment Analysis (GSEA) results for the most significantly enriched pathways associated with NOTCH3 expression. The dot plot highlights pathways, with size reflecting the number of genes and color indicating statistical significance. **(C)** Results of the Reactome pathway analysis for NOTCH3, showing enriched pathways relevant to its function. **(D)** Correlation plots between NOTCH3 and selected genes in different cancer types, indicating potential interactions. Each plot displays the correlation coefficient and *p*-value. **(E–G)** Violin plots comparing NOTCH3 expression between tumor and normal tissues for three different cancer types, with *p*-values denoting statistical significance. **(H)** Kaplan-Meier survival curve stratified by NOTCH3 expression levels. High expression is associated with poorer overall survival, as indicated by the log-rank *p*-value. **(I)** Boxplot comparing the immune cell infiltration between groups with high and low NOTCH3 expression. **(J–N)** Scatter plots showing correlation analysis between NOTCH3 expression and specific immune cell markers. **(O–R)** Immunohistochemical staining of NOTCH3 in different cancer tissue samples, revealing the localization and expression intensity of the protein.

Analysis of NOTCH3-4 expression profiles in colon cancer (Figure 2A) identified high expression of NOTCH3 as significantly associated with poor prognosis. Gene Ontology (GO) and KEGG pathway enrichment analyses of differentially expressed genes (DEGs) between high and low NOTCH3 expression groups revealed strong correlations between NOTCH3 and processes such as extracellular matrix organization, leukocyte migration, cell adhesion, and the PI3K-Akt signaling pathway (Figures 2B, C). Further investigation into NOTCH3 expression in colon adenocarcinoma (COAD) showed significant positive correlations with CD4^+^ T cells, macrophages, neutrophils, and dendritic cells (Figure 2D). Patients with high NOTCH3 expression exhibited elevated scores for stromal, immune, and estimate indices (Figures 2E–G), and worse overall prognosis compared to those with low NOTCH3 expression (Figure 2H). Increased infiltration of M0 and M1 macrophages, along with reduced infiltration of activated NK cells, was observed in the high NOTCH3 expression group, potentially explaining the poorer prognosis (Figure 2I). Significant positive correlations were identified between NOTCH3 and immune checkpoints CTLA4, CD274, and PDCD1 (Figure 2J–N). Immunohistochemical staining demonstrated a notable increase in NOTCH3 expression in colon cancer tissues compared to normal tissues (Figure 2O–R). In summary, these findings suggest that elevated NOTCH3 expression correlates with poor survival outcomes in colon cancer and is associated with altered immune cell infiltration and immune checkpoint expression, highlighting its potential as a prognostic marker and therapeutic target.

### Molecular docking analysis

**FIGURE 3:**
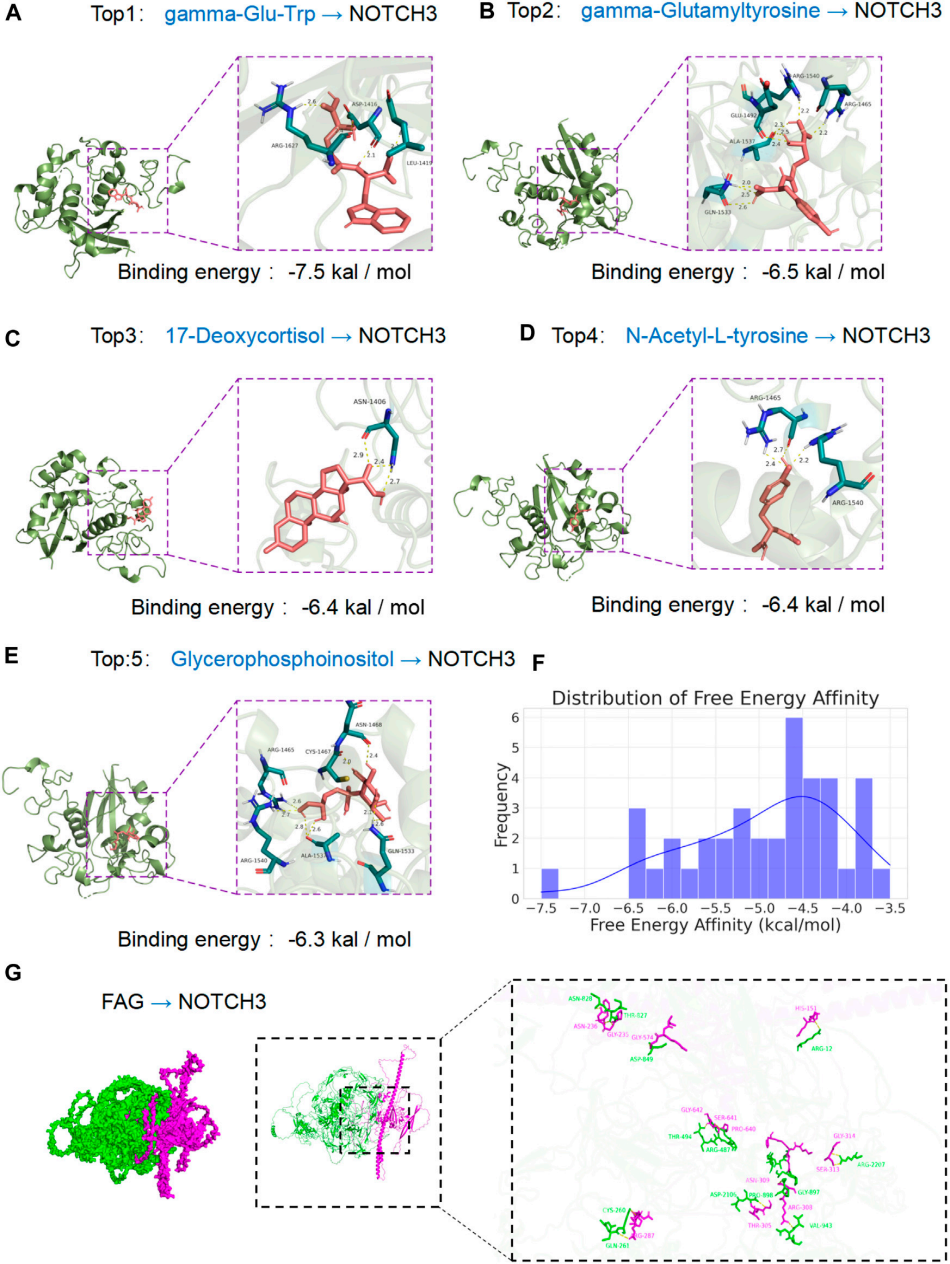
Interaction of various ligands with the NOTCH3 protein assessed by molecular docking. **(A)** Gamma-Glu-Trp–NOTCH3 complex: Depicts the molecular docking position of gamma-Glu-Trp with NOTCH3, demonstrating the lowest binding energy of −7.5 kcal/mol. The panel shows the ligand in a detailed pose, indicating strong affinity and significant biological interaction within the active site of the protein. **(B)** Gamma-Glutamyltyrosine–NOTCH3 interaction: Illustrates the docking of gamma-Glutamyltyrosine with a binding energy of −6.5 kcal/mol. The complex’s interaction points are marked, emphasizing stability and specificity. **(C)** 17-Deoxycortisol–NOTCH3 docking: Shows 17-Deoxycortisol bound to NOTCH3 with a binding energy of −6.4 kcal/mol. The visualization captures the molecular fit within the protein’s binding pocket, highlighting key hydrogen bonds and interaction sites. **(D)** N-Acetyl-L-tyrosine–NOTCH3 binding: Presents the docking conformation of N-Acetyl-L-tyrosine with a binding energy of −6.4 kcal/mol. The figure details the ligand orientation and amino acid residues involved in interaction with the NOTCH3 receptor. **(E)** Glycerophosphoinositol–NOTCH3 interaction: Portrays Glycerophosphoinositol in its binding pose with NOTCH3, showing a binding energy of −6.3 kcal/mol. Specific interactions and binding orientation are detailed, providing insights into the ligand’s mode of action. **(F)** Distribution of free energy affinity: Histogram representing the frequency distribution of binding energies for all ligands tested against NOTCH3. This graph provides an overview of the binding affinity landscape, illustrating the diversity of interactions and highlighting the top-performing ligands as indicated in panels A–E. **(G)** The docking analysis of FGA and NOTCH3 revealed an interface area of 3,717.2 Å^2^, indicating a significant contact region between the two proteins. Detailed examination of the docking interface showed the formation of 22 hydrogen bonds, which contribute to the stability of the protein-protein interaction.

We downloaded 39 chemical compounds from the PubChem database via their CID numbers and docked them sequentially with the NOTCH3 protein for molecular docking analysis. Binding energy calculations revealed that the strength of compound-protein binding increased with lower binding energy. The top five chemical compounds displaying the tightest binding to the NOTCH3 protein were gamma-Glu-Trp (CID 3989307), gamma-Glutamyltyrosine (CID 94304), 17-Deoxycortisol (CID 5753), N-Acetyl-L-tyrosine (CID 68310), and Glycerophosphoinositol (CID 167512). Detailed docking analysis showed that gamma-Glu-Trp exhibited the lowest binding energy (−7.5 kcal/mol) (Figure 3A). Other compounds had binding energies of −6.5 kcal/mol for gamma-Glutamyltyrosine (Figure 3B), −6.4 kcal/mol for 17-Deoxycortisol (Figure 3C), −6.4 kcal/mol for N-Acetyl-L-tyrosine (Figure 3D), and −6.3 kcal/mol for Glycerophosphoinositol (Figure 3E). The distribution of binding energies across all tested compounds, presented in a histogram (Figure 3F), revealed a broad range, illustrating the diversity of interactions with the NOTCH3 protein. The molecular docking results indicate that gamma-Glu-Trp, gamma-Glutamyltyrosine, and 17-Deoxycortisol have the strongest affinities for NOTCH3, potentially offering insights into exercise-induced metabolic shifts. The docking analysis of FGA and NOTCH3 revealed an interface area of 3,717.2 Å^2^, indicating a significant contact region between the two proteins. The change in Gibbs free energy (ΔiG) during docking was calculated to be −18.1 kcal/mol, suggesting an energetically favorable binding interaction. Detailed examination of the docking interface showed the formation of 22 hydrogen bonds, which contribute to the stability of the protein-protein interaction (Figure 3G).

### Effects of glycerophosphoinositol on FGA and NOTCH3 expression

**FIGURE 4:**
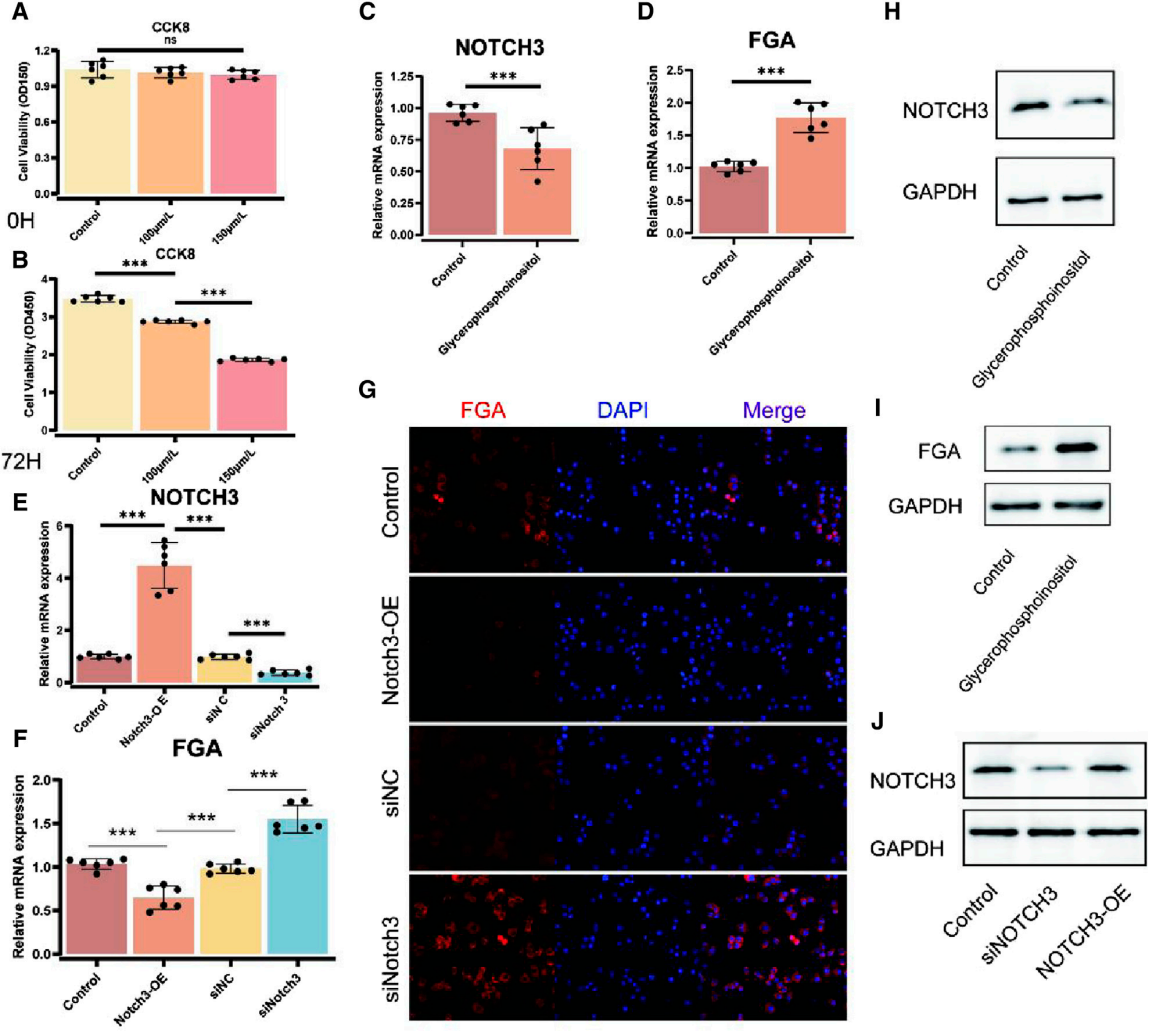
Effects of glycerophosphoinositol on FGA and NOTCH3 expression. **(A, B)** Cell viability tests (CCK-8) were performed at 24 h **(A)** and 72 h **(B)** to survey the impact of glycerophosphoinositol treatment. The results demonstrate changes in cell proliferation after treatment with diverse concentrations of glycerophosphoinositol, indicating significant impacts on cell viability at 72 h (*p* < 0.001). **(C)** qPCR investigation of NOTCH3 mRNA expression after glycerophosphoinositol treatment, uncovering an upregulation of NOTCH3, with a noteworthy *p*-value (*p* < 0.001). **(D)** qPCR investigation of FGA mRNA expression following glycerophosphoinositol treatment, showing expanded FGA expression, with a noteworthy *p*-value (*p* < 0.001). **(E, F)** qPCR validation of NOTCH3 overexpression (NOTCH3-OE) and siRNA models to evaluate the viability of genetic modulation procedures. The results demonstrate significant changes in NOTCH3 **(E)** and FGA **(F)** expression levels under diverse conditions, with *p*-values indicating statistical significance (*p* < 0.001). **(G)** Immunofluorescence images showing FGA expression (red) after NOTCH3 overexpression (NOTCH3-OE) or knockdown (siNOTCH3), with nuclear counterstaining using DAPI (blue). Merged images illustrate the co-localization of FGA and nuclear signals under various experimental conditions, confirming changes in FGA expression visually. **(H)** Western blot analysis of NOTCH3 expression after glycerophosphoinositol treatment, using GAPDH as a loading control. **(I)** Western blot analysis of FGA expression after glycerophosphoinositol treatment, using GAPDH as a loading control. **(J)** Western blot analysis of NOTCH3 expression after NOTCH3 overexpression (NOTCH3-OE) or knockdown (siNOTCH3), using GAPDH as a loading control.

To investigate the impact of glycerophosphoinositol on cell viability, we performed CCK-8 assays at 24 h and 72 h. The results demonstrated changes in cell proliferation after treatment with diverse concentrations of glycerophosphoinositol, indicating significant impacts on cell viability at 72 h (*p* < 0.001) (Figures 4A,B). Specifically, cell viability significantly increased in a concentration-dependent manner upon glycerophosphoinositol treatment. To elucidate the molecular mechanisms underlying these effects, we assessed the expression levels of NOTCH3 and FGA mRNA using qPCR. Our findings revealed that glycerophosphoinositol treatment significantly upregulated NOTCH3 mRNA expression (*p* < 0.001) (Figure 4C). Similarly, FGA mRNA expression was markedly elevated following glycerophosphoinositol treatment (*p* < 0.001) (Figure 4D). Further validation of NOTCH3 overexpression (NOTCH3-OE) and siRNA-mediated knockdown models was performed using qPCR. The results confirmed significant changes in NOTCH3 and FGA expression levels across different conditions, demonstrating the effectiveness of the genetic modulation procedures (*p* < 0.001) (Figures 4E, F). Immunofluorescence staining was conducted to visualize FGA expression after NOTCH3 overexpression (NOTCH3-OE) or knockdown (siNOTCH3). The images revealed FGA expression (red) with nuclear counterstaining using DAPI (blue), and merged images illustrated the co-localization of FGA and nuclear signals under various experimental conditions. These results visually confirmed changes in FGA expression (Figure 4G). Western blot analysis further supported these findings. NOTCH3 expression was significantly upregulated after glycerophosphoinositol treatment compared to controls, with GAPDH serving as a loading control (Figure 4H). Similarly, FGA expression was significantly increased following glycerophosphoinositol treatment, as demonstrated by Western blot analysis (Figure 4I). Additionally, Western blot analysis of NOTCH3 expression after NOTCH3 overexpression (NOTCH3-OE) or knockdown (siNOTCH3) confirmed the effectiveness of these genetic interventions (Figure 4J).

### Impact of NOTCH3 and FGA on cell proliferation and apoptosis

**FIGURE 5:**
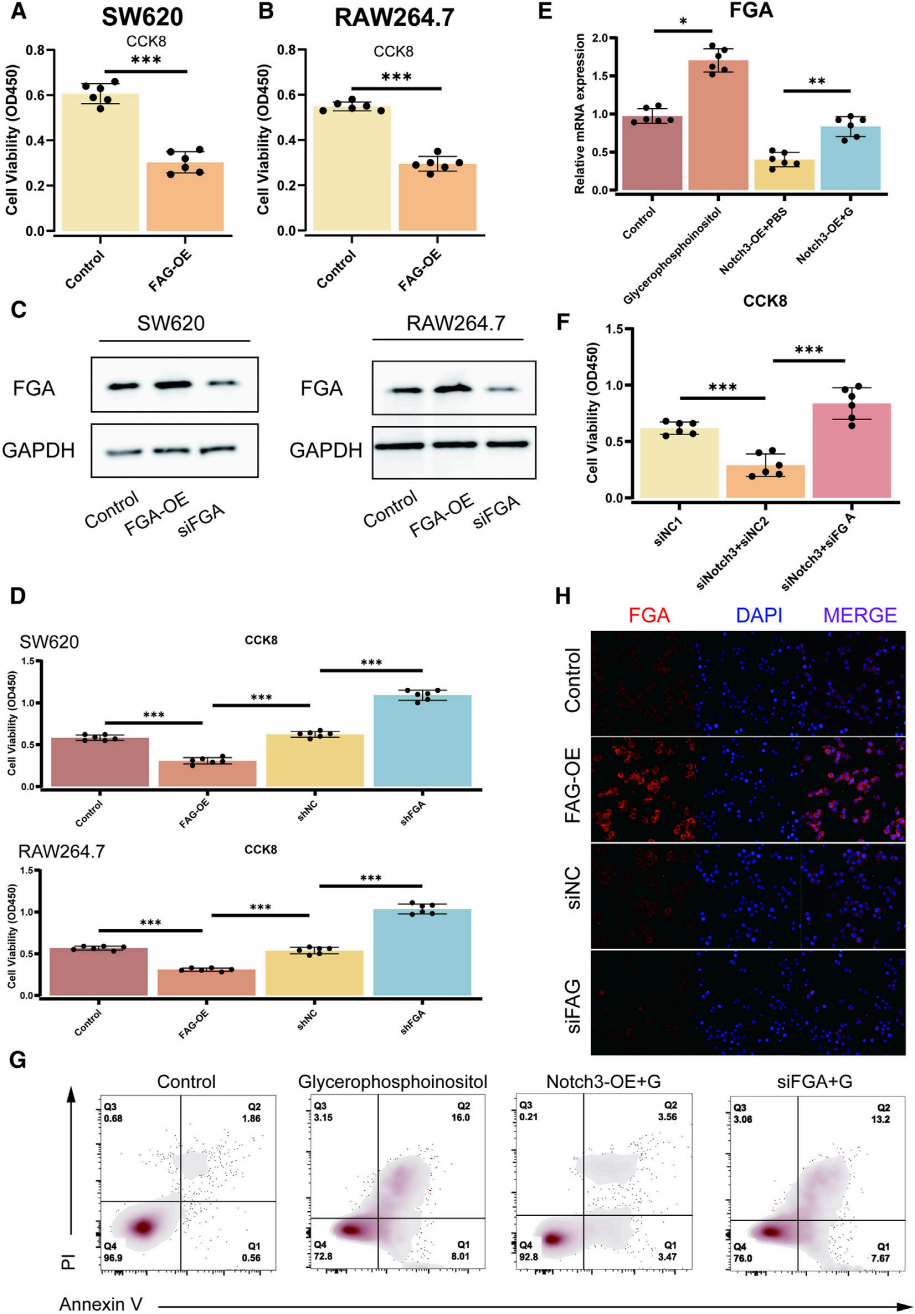
Effect of NOTCH3 and FGA on cell proliferation and apoptosis. **(A, B)** CCK-8 assays validate the FGA-OE model by evaluating changes in cell viability in SW620 and RAW264.7 cell lines. The results demonstrate increased cell viability in both cell lines upon FGA overexpression (FGA-OE) compared to controls, with significant *p*-values (*p* < 0.001). **(C)** Western blot analysis of FGA expression in SW620 and RAW264.7 cells, showing FGA overexpression (FGA-OE) and knockdown (siFGA). GAPDH is used as a loading control. **(D)** CCK-8 assays confirm the effects of FGA overexpression (FGA-OE) and FGA knockdown (siFGA) on cell proliferation in SW620 and RAW264.7 cells. The assays show enhanced proliferation with FGA-OE and diminished proliferation with siFGA, with significant *p*-values (*p* < 0.001). **(E)** qPCR investigation of FGA mRNA levels after NOTCH3 overexpression (NOTCH3-OE), glycerophosphoinositol treatment, or their combination. The results show significant changes in FGA expression, with *p*-values indicating statistical significance (*p* = 0.022 and *p* = 0.005). **(F)** CCK-8 results show the impact of altering NOTCH3 and FGA expression on cell proliferation. The assays reveal a direct correlation between NOTCH3 downregulation, decreased FGA expression, and increased cell proliferation, with significant *p*-values (*p* < 0.001). **(G)** Flow cytometry analysis of apoptosis after NOTCH3 overexpression, glycerophosphoinositol treatment, or siFGA in conjunction with glycerophosphoinositol. Scatter plots depict the apoptosis rates under various conditions, highlighting the effects on cell survival. (H) Immunofluorescence staining demonstrates FGA expression under various conditions (NOTCH3 overexpression or silencing).

The impact of NOTCH3 and FGA on cellular proliferation and apoptosis has been investigated using various assays. CCK-8 assays validate the FGA overexpression (FGA-OE) version through assessing modifications in cellular viability in SW620 and RAW264.7 cell lines. The results confirmed a significant increase in cellular viability in each cell line upon FGA-OE compared to controls, with *p*-values much less than 0.001 (Figures 5A, B). Further CCK-8 assays showed the effects of FGA-OE and FGA knockdown (siFGA) on cellular proliferation in SW620 and RAW264.7 cells, showing enhanced proliferation with FGA-OE and reduced proliferation with siFGA, both appearing noteworthy *p*-values (*p* < 0.001) (Figures 5C, D). qPCR analysis measured FGA mRNA levels post-NOTCH3 overexpression (NOTCH3-OE), glycerophosphoinositol treatment, or their combination, revealing critical changes in FGA expression, with *p*-values demonstrating statistical significance (*p* = 0.022 and *p* = 0.005) (Figure 5E). CCK-8 assays also evaluated the impact of altering NOTCH3 and FGA expression on cell proliferation. The results revealed a direct correlation between NOTCH3 downregulation, decreased FGA expression, and increased cell proliferation, with significant *p*-values (*p* < 0.001) (Figure 5F). Flow cytometry analysis was employed to assess apoptosis post-NOTCH3 overexpression, glycerophosphoinositol treatment, or siFGA in conjunction with glycerophosphoinositol, depicting apoptosis rates under different conditions and significant effects on cell survival (Figure 5G). Immunofluorescence staining visualized FGA localization and expression under different conditions, including NOTCH3 overexpression or silencing, with merged images illustrating co-localization of FGA and nuclear signals. The adjacent bar plots quantified fluorescence intensity, indicating significant changes in FGA expression, with increased expression in the NOTCH3-OE group and decreased expression in the siNOTCH3 group (Figure 5H). These results collectively demonstrate that NOTCH3 and FGA significantly influence cell proliferation and apoptosis.

### Copy number variation and methylation analysis of FGA and NOTCH3 in multiple cancers

**FIGURE 6:**
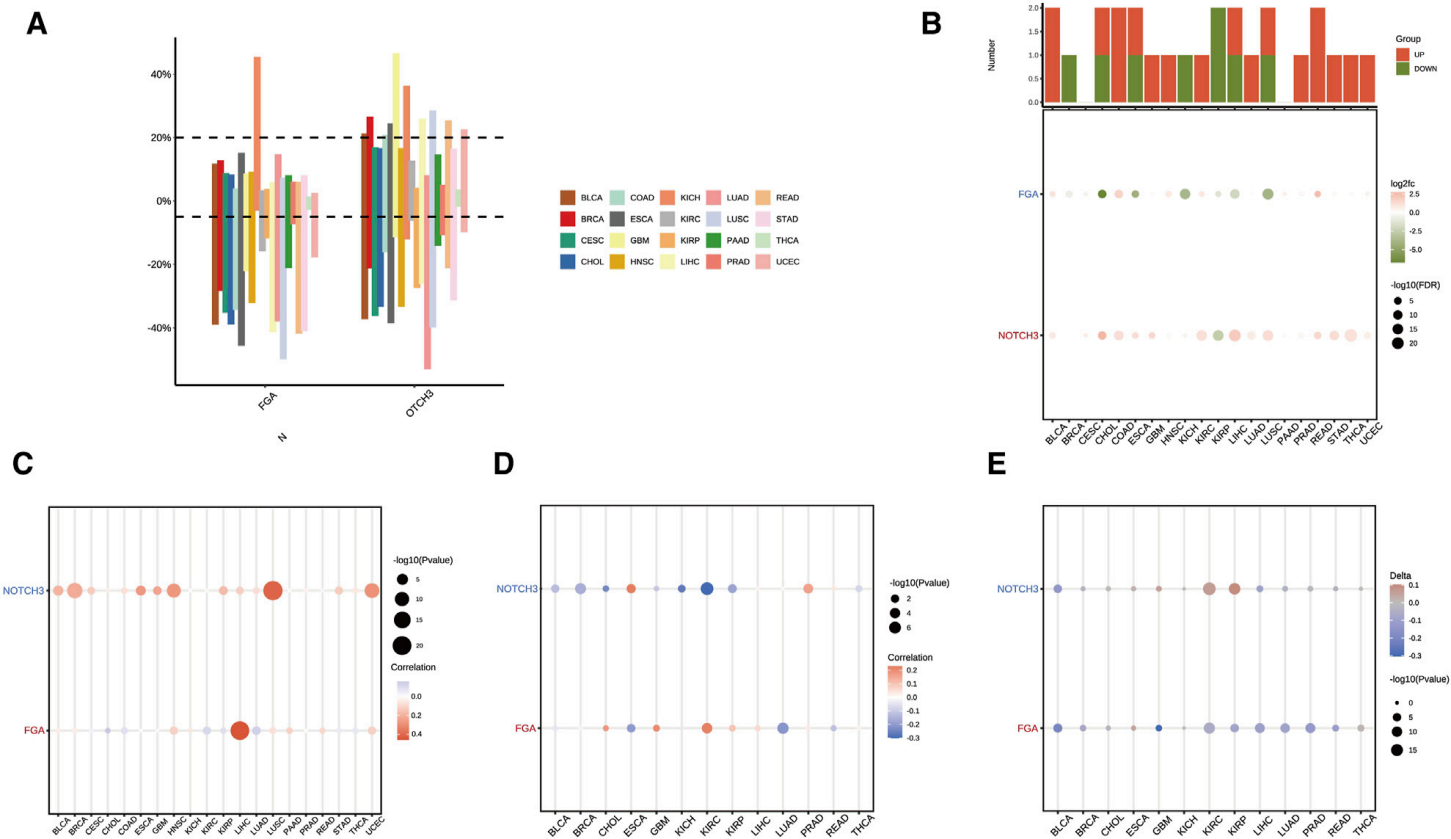
Copy number variation (CNV) and methylation analysis of FGA and NOTCH3 across multiple cancers. **(A)** Distribution of copy number variation (CNV) rates of the FGA and NOTCH3 genes across 20 different cancer types. The bar graph shows the CNV differences between amplification and deletion. **(B)** Differential expression of FGA and NOTCH3 across various cancer types. The top bar plot indicates overall gene expression changes, while the dot plot below shows log fold changes and statistical significance of individual changes. **(C)** Correlation between CNV and expression levels of FGA and NOTCH3 in multiple cancer types. Dot size represents the strength of the correlation, while the color scale denotes the direction (positive or negative) of the correlation. **(D)** Correlation between promoter methylation and gene expression levels of FGA and NOTCH3 across different cancers. Dot size and color indicate the magnitude and direction of the correlation, respectively. **(E)** Difference (delta value) in promoter methylation between tumor and normal tissues for FGA and NOTCH3. Higher delta values represent greater differences between cancer and normal tissues.

Analysis of copy number variation (CNV) in FGA and NOTCH3 across 20 different cancer types revealed distinct CNV rates. The bar graph illustrates differential amplification and deletion patterns among cancer types (Figure 6A). Further differential expression analysis of FGA and NOTCH3 across these cancers highlighted significant gene expression differences, with overall changes shown in a bar plot and specific changes depicted in a dot plot (Figure 6B). The correlation between CNV and expression levels of FGA and NOTCH3 varied across cancers. The dot sizes indicate the correlation strength, while the color scale shows the correlation direction, either positive or negative (Figure 6C). The promoter methylation status was also assessed to understand its relationship with gene expression. Correlations between promoter methylation and expression levels of FGA and NOTCH3 were visualized using dots of varying sizes and colors to reflect the magnitude and direction of these correlations (Figure 6D). Additionally, the difference in promoter methylation between tumor and normal tissues (delta value) was calculated for FGA and NOTCH3, with higher delta values indicating greater differences between the cancerous and normal tissues, thus highlighting specific promoter methylation changes during cancer progression (Figure 6E). These analyses provide insights into how CNV and methylation variations influence the expression of FGA and NOTCH3 across various cancer types, potentially contributing to differential tumor behavior and aiding in identifying new therapeutic targets.

### Correlation between FGA and NOTCH3 expression and tumor prognosis

**FIGURE 7:**
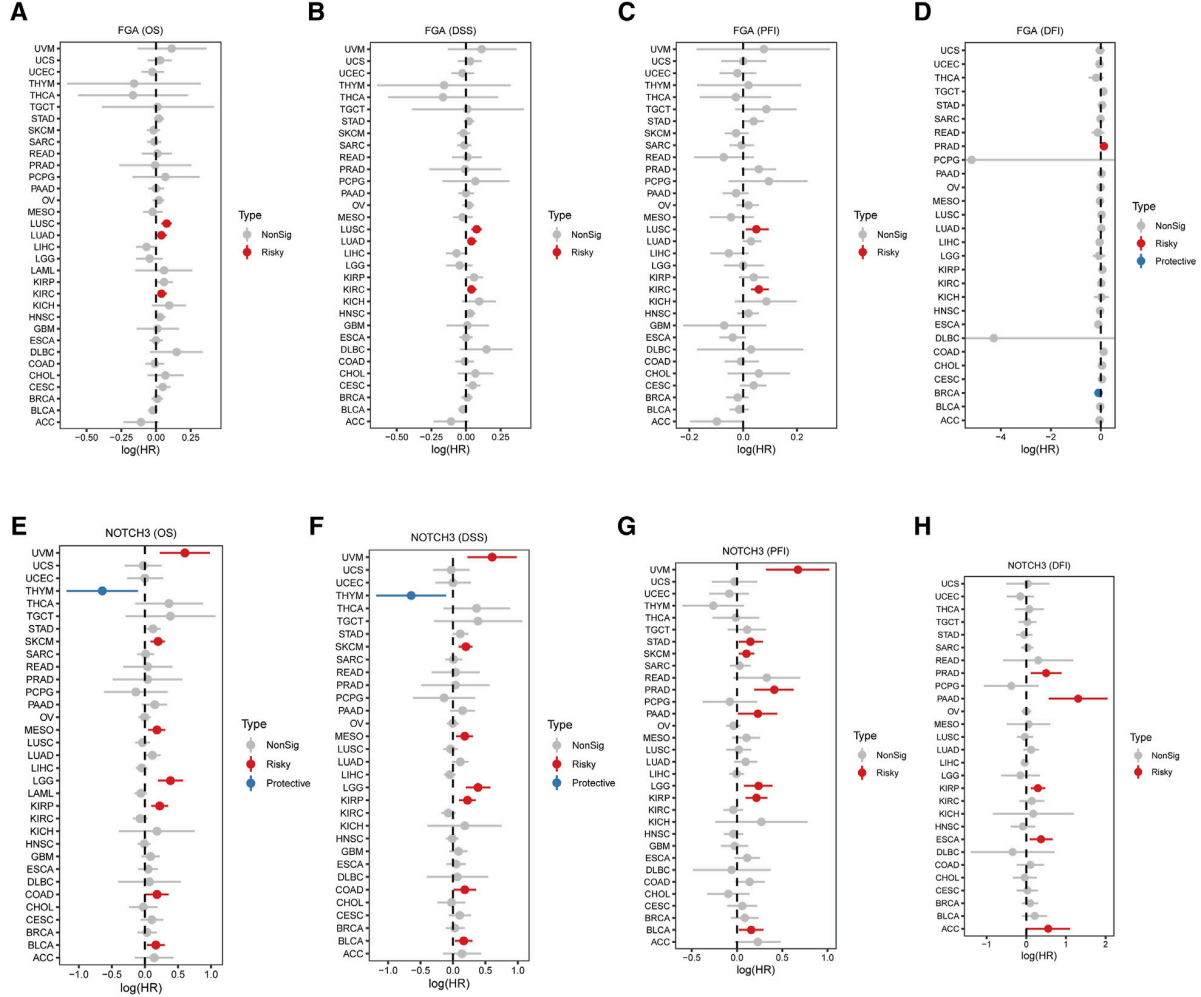
Correlation between FGA and NOTCH3 expression and tumor prognosis across various cancers. **(A–D)** Association between FGA expression and survival metrics in multiple cancer types: Overall survival (OS) correlation with FGA expression, with each point representing a different cancer type. Red points indicate significant negative correlation, and grey points indicate no significant correlation **(A)**. Disease-specific survival (DSS) correlation with FGA expression across cancer types **(B)**. Progression-free interval (PFI) correlation with FGA expression, highlighting significant correlations in various cancers **(C)**. Disease-free interval (DFI) correlation with FGA expression, displaying a significant negative correlation in some cancers and no significant correlation in others **(D)**. **(E–H)** Association between NOTCH3 expression and survival metrics in multiple cancer types. Overall survival (OS) correlation with NOTCH3 expression, with blue points indicating significant positive correlation, and grey points indicating no significant correlation **(E)**. Disease-specific survival (DSS) correlation with NOTCH3 expression, with significant correlations highlighted in blue **(F)**. **(G)** Progression-free interval (PFI) correlation with NOTCH3 expression, showing a mix of significant positive and negative correlations. **(H)** Disease-free interval (DFI) correlation with NOTCH3 expression, with significant correlations depicted in blue and non-significant correlations in grey.

The association between FGA expression and survival metrics was evaluated across multiple cancer types. Significant negative correlations were observed between FGA expression and overall survival (OS) (Figure 7A), disease-specific survival (DSS) (Figure 7B), and progression-free interval (PFI) (Figure 7C), with cancer-specific correlations illustrated. In some cancers, FGA expression showed a significant negative correlation with disease-free interval (DFI), while in others, no significant correlation was detected (Figure 7D). Similarly, correlations between NOTCH3 expression and survival outcomes were analyzed. Significant positive correlations were found between NOTCH3 expression and OS(Figure 7E), with DSS correlations highlighted in blue to indicate a strong association (Figure 7F). A mix of significant positive and negative correlations was observed for PFI (Figure 7G). In the case of DFI, noteworthy relationships were portrayed in blue, whereas non-significant relationships were shown in gray (Figure 7H). Overall, these results suggest that FGA and NOTCH3 expression levels are strongly linked to various survival outcoomes across different cancers. The analysis reveals patterns that could help identify prognostic markers and stratify patients based on their risk.

## Discussion

Recent studies have highlighted the significance of the Notch signaling pathway, which includes NOTCH3, in muscle adaptation and its frequent dysregulation in cancers, including COAD (Teoh and Das, 2018; Katoh and Katoh, 2019). Additionally, FGA, a component of fibrinogen, plays a role in the extracellular matrix (ECM) and is involved in remodeling processes associated with both exercise and cancer (Wang et al., 2016; Kim et al., 2022). Understanding the regulatory mechanisms involving FGA and NOTCH3, particularly their post-translational modifications (PTMs), is crucial for identifying new therapeutic targets and prognostic markers (Alqudah et al., 2013; Kang et al., 2021). Our study provides a comprehensive investigation of the roles of Fibroleukin (FGA) and NOTCH3 in exercise-induced muscle adaptation and COAD progression (Gopalakrishnan et al., 2014). Through the identification of differentially expressed genes (DEGs) and analysis of the Notch signaling pathway, we have established a significant connection between these proteins and the organic results of intrigued (Talora et al., 2008; Mummery-Widmer et al., 2009). Our results indicate an association between NOTCH3 expression and poor prognosis in COAD, suggesting its involvement in cancer cell survival and proliferation through interaction with the PI3K-Akt pathway. Moreover, the modulation of FGA and NOTCH3 expression by glycerophosphoinositol suggests potential implications for targeted treatments.

The identification of 114 DEGs in striated muscle and 31 in the colon group underscores the complexity of the molecular changes initiated by exercise. The consistent upregulation of the Notch signaling pathway in exercise samples suggests a potential role for NOTCH3 in muscle adaptation (Baeten and Lilly, 2015). The association between high NOTCH3 expression and poor COAD prognosis is a significant finding, consistent with previous studies implicating Notch signaling in cancer progression. Fibronectin Leucine-Rich Transmembrane Protein 2 (Fibroleukin, FGA) and NOTCH3, known for their roles in muscle adptation and cancer progression, are particularly intriguing in this context. Current literature suggests that FGA and NOTCH3 are essential to both muscle adaptation and CRC pathophysiology, although their precise functions and interactions in these processes are not fully understood. FGA, as a component of the extracellular matrix, has been implicated in muscle recovery and fibrosis, while NOTCH3, a transmembrane receptor, is involved in cell differentiation and proliferation. Research indicates that inhibiting NF-κB signaling may intersect with the NOTCH3 pathway in cancer progrerssion (Kontomanolis et al., 2018). In our study, both overall survival (OS) and disease-specific survival (DSS) were included to provide a comprehensive view of patient outcomes related to FGA and NOTCH3 expression across different tumor types. OS encompasses all causes of death, offering a broad measure of survival, while DSS specifically targets deaths attributable to the investigated disease, thus providing a more focused perspective on the disease’s impact. Specifically, in certain tumor types, high FGA expression was associated with poorer OS and DSS, indicating its potential role as a negative prognostic marker. Conversely, the expression of NOTCH3 appeared to have varying impacts on survival depending on the tumor type, with potential protective or harmful effects. The differences in OS and DSS correlations underscore the importance of considering multiple survival metrics when evaluating prognostic factors. While OS provides insights into overall patient health and longevity, DSS offers a clearer view of the direct impact of the disease, free from confounding variables such as comorbidities or treatment-related complications. This dual approach allows for a more nuanced understanding of the biological mechanisms underlying tumor progression and patient survival. Our findings suggest that targeted therapies regulating FGA and NOTCH3 expression could improve patient prognosis based on specific tumor types and survival conditions. Future research should focus on elucidating the molecular pathways through which FGA and NOTCH3 influence tumor biology and patient survival and validate these biomarkers in larger independent cohorts.

The impact of high-pressure oxygen on the Notch signaling pathway following severe carbon monoxide poisoning in mice provides insights into the role of NOTCH3 in disease progression (Hu et al., 2023). Exercise offers numerous benefits, such as improving cerebral blood flow and functional outcomes in patients with vascular cognitive impairment and dementia (Karamacoska et al., 2023; Khan et al., 2023), providing insights into how exercise affects the overall health of CRC patients. Understanding the definition of a hypertension-like response in normal individuals is useful, as it relates to exercise adaptation and provides context for the interactions between exercise, muscle health, and CRC progression (Laukkanen and Kunutsor, 2021; Guo et al., 2022). Promoting a healthy lifestyle among cancer patients and their families and examining exercise-induced muscle adaptation provides new perspectives on exercise and metabolic health (Huang et al., 2018; Maddocks, 2020; Hou et al., 2022). Additionally, low-intensity pulsed ultrasound promoting skeletal muscle regeneration demonstrates the potential for muscle recovery physiotherapy (Abrunhosa et al., 2011; Qin et al., 2023). Exercise reduces IGF1R aggregation, alleviating neuroinflammation in transgenic mice (Chen et al., 2024). Mesenchymal stem cell-derived extracellular vesicles target the let-7a/Tgfbr1 axis, suggesting potential therapeutic benefits for muscle adaptation (Wang P. et al., 2023). Finally, brain metabolomics is important for advancing our understanding of the underlying biological processes (He et al., 2023). PRMT5 promotes tumor metastasis by methylation-activated AKT (Huang et al., 2022).

The importance of transcriptomics research lies in its ability to provide new insights into cellular heterogeneity in complex biological processes (Wu et al., 2023). Advances in transcriptomics have offered new perspectives on understanding disease onset and progression (Li et al., 2022). The role of transcriptomics in revealing the immune microenvironment is crucial for the diagnosis and prognosis of various diseases (Yang K. et al., 2023; Yang Y. et al., 2023). In modern medical research, drug therapy and bioinformatics analysis methods have become important research tools. The correlation between FGA/NOTCH3 expression and survival metrics, with adverse correlations for FGA and favorable for NOTCH3, provides valuable insights into the prognostic significance of these proteins. These findings align with previous research that has linked NOTCH3 overexpression with unfavorable outcomes in various cancers. While our study offers a robust analysis of the roles of FGA and NOTCH3, there are limitations that must be acknowledged. The sample size for the gene expression and cellular assays may limit the generalizability of our findings. Additionally, the *in vitro* nature of the cellular viability and apoptosis assays means that the effects of glycerophosphoinositol on FGA and NOTCH3 expression *in vivo* need to be confirmed. Further research is needed to fully understand the mechanisms by which FGA and NOTCH3 contribute to exercise-induced muscle adaptation and COAD progression (Wang et al., 2008; MacKenzie et al., 2013). Longitudinal research that tunes adjustments in FGA and NOTCH3 expression in reaction to workout and within the context of COAD ought to provide greater definitive proof of their roles (Yuan et al., 2017).

The use of bioinformatics databases, through large-scale data analysis, has revealed the relationship between specific physiological indicators and long-term prognosis, emphasizing its application value in clinical decision-making (Chen et al., 2022a; Chen et al., 2022b; Du and Liu, 2024; Yao et al., 2024). The molecular docking results, which identified gamma-Glu-Trp, gamma-Glutamyltyrosine, and 17-Deoxycortisol as strong NOTCH3 binders, provide a starting point for the development of small molecule inhibitors targeting the NOTCH pathway. The impact of glycerophosphoinositol on FGA and NOTCH3 expression, as well as its effects on cellular viability and apoptosis, support the potential therapeutic application of this compound in COAD treatment. However, our study extends current knowledge by elucidating the specific molecular mechanisms and interactions involved. The identification of the NOTCH signaling pathway as a commonly upregulated pathway in exercise samples is a novel observation that warrants further investigation. Furthermore, the findings of this study have significant implications for the clinical management of COAD and the development of exercise-based interventions for cancers patients. The identification of FGA and NOTCH3 as potential prognostic markers could guide patient stratification and treatment decisions.

## Conclusion

In conclusion, our research contributes to a deeper information of the molecular mechanisms underlying exercise-induced muscle adaptation and COAD progression. The identification of FGA and NOTCH3 as potential prognostic markers and therapeutic targets opens new avenues for future research and clinical application. Further studies are needed to fully elucidate the roles of these proteins and to translate these findings into effective strategies for cancer prevention and treatment.

## Data Availability

The datasets presented in this study can be found in online repositories. The names of the repository/repositories and accession number(s) can be found in the article/Supplementary Material.
